# In vitro matured oocytes have a higher developmental potential than in vivo matured oocytes after hormonal ovarian stimulation in *Callithrix jacchus*

**DOI:** 10.1186/s13048-024-01441-0

**Published:** 2024-06-01

**Authors:** Olena Y. Tkachenko, Tobias Kahland, Dimitri Lindenwald, Michael Heistermann, Charis Drummer, Maria Daskalaki, Nancy Rüger, Rüdiger Behr

**Affiliations:** 1 Platform Degenerative Diseases, German Primate Center—Leibniz Institute for Primate Research, Kellnerweg 4, 37077 Göttingen, Germany; 2 Endocrinology Laboratory, German Primate Center—Leibniz Institute for Primate Research, Kellnerweg 4, 37077 Göttingen, Germany

**Keywords:** Marmoset, Ovary, Hormonal stimulation, hCG, In vivo matured oocytes, Embryo outcome, IVM

## Abstract

**Background:**

The common marmoset, *Callithrix jacchus*, is an invaluable model in biomedical research. Its use includes genetic engineering applications, which require manipulations of oocytes and production of embryos in vitro. To maximize the recovery of oocytes suitable for embryo production and to fulfil the requirements of the 3R principles to the highest degree possible, optimization of ovarian stimulation protocols is crucial. Here, we compared the efficacy of two hormonal ovarian stimulation approaches: 1) stimulation of follicular growth with hFSH followed by triggering of oocyte maturation with hCG (FSH + hCG) and 2) stimulation with hFSH only (FSH-priming).

**Methods:**

In total, 14 female marmosets were used as oocyte donors in this study. Each animal underwent up to four surgical interventions, with the first three performed as ovum pick-up (OPU) procedures and the last one being an ovariohysterectomy (OvH). In total, 20 experiments were carried out with FSH + hCG stimulation and 18 with FSH-priming. Efficacy of each stimulation protocol was assessed through in vitro maturation (IVM), in vitro fertilization (IVF) and embryo production rates.

**Results:**

Each study group consisted of two subgroups: the in vivo matured oocytes and the oocytes that underwent IVM. Surprisingly, in the absence of hCG triggering some of the oocytes recovered were at the MII stage, moreover, their number was not significantly lower compared to FSH + hCG stimulation (2.8 vs. 3.9, respectively (ns)). While the IVM and IVF rates did not differ between the two stimulation groups, the IVF rates of in vivo matured oocytes were significantly lower compared to in vitro matured ones in both FSH-priming and FSH + hCG groups. In total, 1.7 eight-cell embryos/experiment (OPU) and 2.1 eight-cell embryos/experiment (OvH) were obtained after FSH + hCG stimulation vs. 1.8 eight-cell embryos/experiment (OPU) and 5.0 eight-cell embryos/experiment (OvH) following FSH-priming. These numbers include embryos obtained from both in vivo and in vitro matured oocytes.

**Conclusion:**

A significantly lower developmental competence of the in vivo matured oocytes renders triggering of the in vivo maturation with hCG as a part of the currently used FSH-stimulation protocol unnecessary. In actual numbers, between 1 and 7 blastocysts were obtained following each FSH-priming. In the absence of further studies, FSH-priming appears superior to FSH + hCG stimulation in the common marmoset under current experimental settings.

**Supplementary Information:**

The online version contains supplementary material available at 10.1186/s13048-024-01441-0.

## Introduction

The common marmoset, or white-tufted-ear marmoset (*Callithrix jacchus*), is a non-endangered, classified as least concern, New World monkey species. Being native to the northeastern Brazil, the common marmoset has become a valuable laboratory animal model worldwide. Research colonies keeping common marmosets imported the animals from their native habitat in 1960–70-ies. These colonies now exist in Europe (France, Germany), UK, Northern America (USA and Canada), Southern America (Brazil), Asia (Israel, China and Japan) and Australia [1]. *C.jacchus* is used as a model species in a wide range of biomedical disciplines, including, but not limited to, research in neurologic, inflammatory and infectious diseases, aging, obesity, reproductive biology, behavioural science and in preclinical testing [2, 3].

Several specific reproductive characteristics make the common marmoset a highly advantageous non-human primate (NHP) research model. Common marmoset females, same as other callitrichids, have the highest lifetime reproductive potential among non-human primates. This is due to a relatively early attainment of sexual maturity (the average age at the first conception is around 2.5 years), multiple infants per litter (naturally 2), an unusual for primates postpartum ovulation and therefore short inter-birth intervals, and absence of menopause [4].

Notwithstanding the considerable phylogenetical distance between callitrichids and the Old World primates, marmosets still display a high genetic similarity to humans compared with non-primate species and are readily used in reproductive and genetic modification studies as a human model [5, 6]. Similarly to humans and crab-eating macaques [7], the average ovarian cycle length in marmosets is 28 days and exhibits no seasonality [8, 9], as, for example, seen in rhesus monkeys, which are also commonly used as human models. However, marmosets also feature some substantial differences to Old World primates and humans. In contrast to humans, the average follicular phase in marmosets is considerably shorter (8 days [8, 9] compared to 16.9 days in humans [10]), while the luteal phase is longer than in humans (on average, 20 days in marmoset [8, 9] vs. 12.4 days in humans [10]) and more variable. Next, preimplantation and early post-implantation embryo development is much slower in marmosets than in the Old World primates: the embryo implantation can occur as early as on day 6 post ovulation in human [11], on day 9 in rhesus [12], yet only on day 11–13 in marmosets [13]. Further, the transition from embryonic to fetal stage of development, defined as the completion of organogenesis, takes place at around day 53–58 (of 266 days of gestation) in humans [14], day 48 (of 160 days) in rhesus [15], yet only at day 73–87 (of 144 days) in marmosets [16].

The reproductive endocrinology of marmosets and other New World monkey species differs from the Old World primates’ as well. In contrast to the latter, New World monkeys possess a special type of LH receptor (LHR) lacking exon 10, known as LHR type II [17]. In humans and other primates, LHR is activated by two hormones – luteinizing hormone (LH) produced in the pituitary and chorionic gonadotropin (CG) produced by embryonic trophoblast cells and by the placenta. These two hormones have an identical α subunit (which is common to the four glycoprotein hormones – FSH, LH, CG and TSH), while the β subunit is unique for each specific hormone [18]. In humans, the β subunit of hCG has an 85% homology with the β subunit of LH [18]. In the common marmoset, however, CG is the only gonadotrophin with luteinising function present in the pituitary gland [19]. Müller et al*.* [19] proposed that during the evolutionary establishment of the New World monkey lineage, when LHR lost its exon 10, CG took over the functions of LH, since it is able to bind to LHR even with its exon 10 missing. According to Müller et al*.* [19], the earlier studies, which analysed LH in the common marmoset [20–23], had actually measured CG levels instead of LH and therefore their results should be reinterpreted [19]. However, marmoset LHR type II might still possess at least residual ability to bind LH, since in the earlier culture systems human FSH (hFSH) was used in combination with human LH (hLH) to trigger oocyte maturation in vitro [24]; but this issue requires additional research.

The increasing use of marmosets in biomedical research, including their genetic modifications, requires manipulations of oocytes and production of embryos in vitro. To maximize the recovery of suitable oocytes, ovarian stimulation is applied. However, compared to humans, relatively little progress has been made in the development of hormonal stimulation approaches in marmosets. Since published marmoset embryo production rates remain low [25–29], further research towards optimisation of ovarian stimulation is needed.

To the best of our knowledge, all protocols of hormonal stimulation for marmosets published up to date, only include application of hFSH and/or hCG in different doses and for a variable duration of time. Most of the stimulation approaches have been summarised in two reviews on assisted reproductive technologies in this species [30, 31]. In brief, up to date there are three basic strategies applied for hormonal stimulation in marmosets: (1) Stimulation with hFSH followed by ovulation triggering with hCG; (2) Ovulation triggering with hCG in the natural cycle; and (3) hFSH-priming. While the first two strategies actually aim to produce in vivo matured oocytes, the latter one is focused exclusively on increasing the number of large antral follicles containing immature cumulus-oocyte complexes (COCs) which are to be subjected to in vitro maturation. However, the only use of FSH-priming was published by Gilchrist et al*.* [32], while all recent (published in the last decade) studies in marmosets involving hormonal stimulation used the protocols with FSH + hCG [29, 33–35], assuming their higher efficiency.

No direct comparison between FSH + hCG and FSH-priming approaches in marmosets has ever been published; in addition, the ovum pick-up (OPU) technique has never been attempted in FSH-priming cycles. The equal or better efficacy of the hFSH-priming approach omitting the in vivo oocyte maturation triggering with hCG would represent an advantage in adherence with the 3Rs principle due to reduction of the total number of injections given per animal and avoidance of potential adverse effects of hCG, such as the ovarian hyperstimulation syndrome [36]. The present study was conducted within a larger project aiming at the production of genetically modified marmoset monkeys (the results will be published separately). Here, we report the relative efficacy of two hormonal stimulation strategies: hFSH + hCG vs. hFSH-priming and the practical consequences resulting from this comparison.

## Materials and methods

### Animals

All procedures involving animals were carried out according to the German Animal Welfare Act, the relevant guidelines and approved by the competent authority, i.e. Niedersächsisches Landesamt für Verbraucherschutz und Lebensmittelsicherheit (LAVES; Animal Experiment Permission #33.19–42,502-04–19/3221). This study was carried out as part of the hormonal stimulation optimization protocol for a larger project consisting of multiple subprojects aiming at the production of genetically modified marmoset monkey model intended for further research.

The animals used in this study are part of the self-sustained research colony of the German Primate Center, which has originally been established in the 70-ties with *C.jacchus* imported from England and which now includes around 480 animals. All animals used for this study were housed according to the applicable legal guidelines and institutional practice of the German Primate Center for *C. jacchus* [5, 32, 37, 38]. Well-being of the animals was controlled daily by animal care personnel with additional regular health check-ups by veterinarians. Marmosets were housed at 25 ± 1 °C and 65 ± 5% relative humidity and air circulation and filtration at artificially controlled light/dark cycle (12/12 h). Vertically-oriented stainless steel cages [165 cm (H) × 65 cm (W) × 80 cm (D)] were furnished with wooden elements for environmental enrichment. Rooms and cages were cleaned with water biweekly. The animals received pelleted primate food Altromin 0633 (Altromin Spezialfutter GmbH & Co. KG, Lage, Germany) ad libitum*.* In addition, approx. 20 g mash (bananas, carrot juice, yogurt and quark enriched with vitamins and minerals) per animal was served in the morning and 30 g fruit or vegetables mixed with rice and gum Arabic were provided in the afternoon. Furthermore, mealworms or locusts were offered three times per week. Total daily vitamin D3 intake provided as a liquid supplement to the morning mash consisted of approx. 140 IU/animal, while additional approx. 150 IU/animal were provided with food pellets. Drinking water was always available in the cages while fruit teas were provided occasionally.

In total, 14 female marmosets were used as oocyte donors in this study (age: from 3.3 to 6.1 years (mean 4.6 ± 0.7 (SD) y), bodyweight: from 371 to 528 g (mean 442 ± 35.5 g)). Some animals have previously undergone one or more OPU procedures, but their oocytes/embryos were cultured under different conditions; therefore, data from these operations were not included in this study. Oocyte donors were housed with male partners to ensure the optimal ovarian function [39]. In addition, 5 males with proven fertility were used as sperm donors (age: from 5.6 to 7.7 years (mean 6.7 ± 0.9 years), bodyweight: from 368 to 561 g (mean 443 ± 39.1 g)). These were housed in male-male pairs to avoid copulations, which could affect the volume of the sample and/or its quality. In our colony, neither homosexual behaviour nor masturbation have been observed of yet (own observations).

### Cycle monitoring and control

Ovarian cycles were monitored by plasma progesterone levels measurements performed twice weekly [9, 40] and carried out by enzyme immunoassay as described earlier [41]. In brief, 0.2 mL blood was collected from the *Vena femoralis* using a 1 mL syringe with a 26G heparin-primed needle. The blood samples were kept on ice and transferred to the endocrinology laboratory, where the samples were centrifuged at 1500 g for 10 min at RT to obtain the plasma for progesterone analysis. Progesterone was determined by a direct, non-extraction EIA using a monoclonal antibody to 11-hydroxyprogesterone-hemisuccinate: BSA (Quidel clone no. 425; CL425) [41]. Progesterone–horseradish peroxidase (HRP) was used as enzyme conjugate in the EIA. Samples were usually measured in a 1:30 dilution in assay buffer (TBS, pH 7.2). The assay has previously been analytically and biologically validated [41], and inter-assay coefficients of variation of high- and low-value quality controls run in each assay were both < 15% across all measurements. Progesterone concentrations < 10 ng mL^−1^ indicated the follicular phase, whereas concentrations > 10 ng mL^−1^ indicated the luteal phase of the ovarian cycle [9, 40].

To time the beginning of hormonal stimulation and to prevent natural pregnancies, luteolysis was induced in oocyte donors by prostaglandin F_2α_ analogue (PGF-2α, Estrumate, Essex Tierarznei, Munich, Germany) given i.m. [38].

On the day following PGF-2α injection, i.e. day 1 of the new cycle, hormonal stimulation was initiated. Injections of 25–35 IU recombinant human FSH (Gonal-f, Merck Europe B.V., Amsterdam, Niederlande) were performed daily at 09:00 with the total duration of FSH-stimulation according to the study protocol (see below). In vivo ovulation was triggered with the injection of 75 IU hCG (Ovogest, MSD Animal Health, Unterschleissheim, Germany) given at 15:00 on the last day of stimulation, and the operation (OPU or ovariohysterectomy (OvH)) was performed 18–20 h later.

### Study design

Two stimulation approaches were investigated in this study: stimulation with hFSH plus in vivo maturation triggering with hCG (hereafter referred to as "FSH + hCG") was compared with priming with human recombinant FSH only (hFSH; hereafter referred to as "FSH-priming"). In total, 20 FSH + hCG stimulation cycles were performed in 11 individual animals. Out of these, 16 stimulations included eight (8) daily injections of FSH, and in four (4) stimulation cycles the duration of the FSH-stimulation was individually adjusted (see below) and varied between 6 and 9 days. Eighteen stimulations of 9 individual animals were performed as FSH-priming for 6–9 days. The stimulation length chosen depended on the individual follicular phase length, which was calculated based on the data from previous cycles. Additionally, individual responsiveness to FSH in previous stimulation cycles was considered. In case of rising progesterone observed on the day of operation, the duration of FSH-stimulation was reduced in the following stimulation cycles. The operations were always planned 1–2 days before the predicted day of the ovulation (Table 1).

**Table 1:** Two hormonal stimulation protocols applied in *Callithrix jacchus* for recovery of oocytes via OPU or ovary dissection

Variables	Hormonal stimulation protocols	
	FSH + hCG	FSH-priming
FSH dose	25 IU	25 or 35 IU
Days of FSH	5.5–9	6–9
hCG dose	75 IU	Not applicable
Total number of stimulations	20	18

**Fig. 1:**
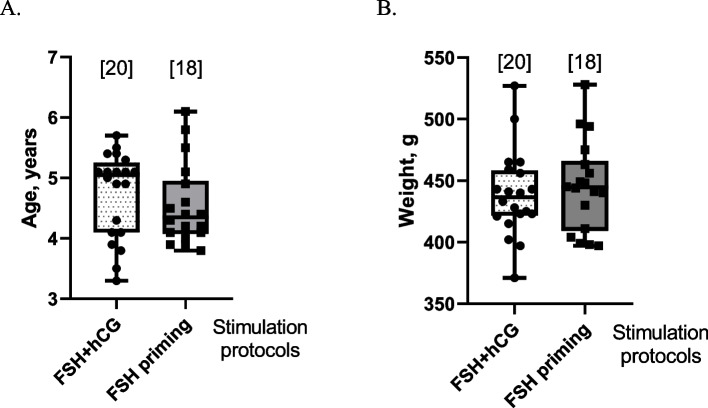
Age (**A**) and bodyweight (**B**) of *C.* *jacchus* females in both study groups. The number of stimulations in each group is shown in square brackets

There were no statistically significant differences in either age or body weight assessed at the moment of each operation between the animals of the two stimulation protocol groups (Fig. 1).

Individual adjustment of the duration of FSH-stimulation was based on the natural follicular phase (FP) length. The average length of the FP was calculated based on several cycles preceding the experiment. In addition, ovarian response to FSH was controlled by ultrasound and in the case of an unexpectedly fast growth of the follicles the current stimulation cycle was cancelled due to logistical reasons and inability to perform unscheduled surgeries. The duration of stimulation was adjusted accordingly in the next stimulation cycle.

### OPU and OvH procedures

For each animal, the oocyte collection was performed in four stimulation cycles: the first three surgeries were OPU procedures, while the fourth surgery was carried out as an OvH. Each animal received Metacam (0.5 mg/mL meloxicam) 0.15 mg p.o. as premedication and 0.1 mg postoperatively over 3–4 days for pain management. In addition, all animals received antimicrobial treatment postoperatively (Hostamox LA 150 mg/mL, 15 mg/animal i.m. immediately post-OP and every second day for 4 days).

All operations were carried out under injection narcosis: to this end, ketamine 100 mg/mL at 25–50 mg/kg, medetomidine 1 mg/mL at 0.05–0.1 mg/kg, and atropine 0.513 mg/mL at 0.05 mg/kg) were administered i.m. For the last operation (OvH of one animal from the FSH-priming group), new narcosis was used, with the aim to improve the general tolerability and to shorten the postoperative recovery/waking-time. The new protocol consisted of alfaxalone 10 mg/mL at 10 mg/kg, midazolam 5 mg/mL at 0.25 mg/animal and buprenorphine 0.3 mg/mL at 0.005–0.025 mg/kg administered i.m., and bupivacaine at 2 mg/kg applied topically. The outcome of the operation carried out with the new narcosis was comparable to the outcomes seen with the old protocol. OPU was performed using an aspirator (Labotect Aspirator 3, Labotect, Göttingen, Germany). 23Gx3/4 puncture needles (BD, Allschwil, Switzerland) were connected to a sterile Heidelberger extension tube (#13,802, Labotect, Göttingen, Germany). The oocytes were collected into a 10 mL tube with pre-warmed (37 °C) handling medium (POE-CM ([Porcine Oocyte/Embryo Collection Medium], CosmoBio, Tokyo, Japan) supplemented with 5% FBS (Biochrom AG, Berlin, Germany) and 2.5 IU/mL heparin (Heparin-Natrium-5000-ratiopharm®, Ratiopharm, Ulm, Germany).

After ovariohysterectomy, the ovaries attached to the uterus were immediately placed into pre-warmed (37 °C) POE-CM + 5% FBS and transferred to the laboratory, where large ovarian follicles were mechanically dissected using 26G needles and/or forceps to recover oocytes.

### Culture plate preparation

All culture steps were performed in 4-well dishes (NUNC, Thermo Fisher Scientific, Osterode am Harz, Germany, Catalogue number #179,830) in drops of the respective culture medium (see below) under light mineral oil (LiteOil®, LifeGlobal Europe, Brussels, Belgium): the drops in the first three wells were used for washing, followed by culturing in the fourth well. All culture plates (for short-term storage; in vitro maturation (IVM); in vitro fertilization (IVF); embryo culture) with drops of medium under pre-incubated light mineral oil were prepared the day before use and stored overnight in an incubator with gas conditions according to the developmental stage to assure proper pH (7.3) and dissolved oxygen concentration.

### Oocyte collection and in vitro maturation

All available cumulus-oocyte complexes (COCs) and naked oocytes retrieved during the OPU procedure or dissected out of antral follicles from the removed ovaries were immediately transferred from the handling medium (see above) into hormone-free POM medium (CosmoBio, Tokyo, Japan) supplied with 5% FBS (Biochrom AG, Berlin, Germany) and left at 37.5 °C, 5.5% CO_2_ in air, until the collection was finished. Only healthy, non-degenerated COCs and oocytes were selected for culture. These were then transferred into culture dishes with 500 µL drops of IVM medium with hormones (POM, 5% FBS, 1 IU/mL FSH and 1 IU/mL hCG) under oil. IVM was performed at 37.5 °C, 5.5% CO_2_ in air, for 29-30 h. The choice of gas conditions has been described in detail in Tkachenko et al*.* [42]. All oocytes and embryos from different donors were always cultured separately.

### Sperm collection and in vitro fertilization

At least two sperm donors were used for each experiment. Sperm was collected by penile vibrostimulation (PVS) procedure described in detail in [43]. Briefly, ejaculation was induced using a FertiCare personal vibrator (Multisept ApS, Rungsted, Denmark) fitted with a 2 cm × 0.5 cm glass tube with rounded edges serving as an artificial vagina. Sperm collection medium (HEPES-buffered Tyrode’s lactate with 0.25 mM sodium pyruvate (Sigma-Aldrich (Merck), Darmstadt, Germany) and 0.3% wt/vol Bovine Serum Albumin ((BSA, Sigma-Aldrich (Merck), Darmstadt, Germany), pH 7.3, 400 µL, 37 °C) was immediately added to the ejaculate to minimize coagulation of the seminal plasma. In the laboratory, sperm samples were washed using a density gradient centrifugation (300 g, 18 min, 37 °C, soft mode) The 40%/80% density gradient prepared with PureSperm solution (PureSpermTM 100, Nidacon, Mölndal, Sweden) and Sperm collection medium. Washed sperm samples were carefully pipetted on the bottom of glass tubes with 600 µL Capacitation medium (Tyrode’s lactate with 0.25 mM sodium pyruvate and 0.3% wt/vol BSA) and placed in the incubator with 37.5 °C, 5% O_2_/5.5% CO_2_ for 40 min for capacitation. Capacitated sperm samples were analysed for motility and the sample with the highest rapid progressive motility was chosen for IVF. On average, samples with concentration of ca. 5 000 000 sperm/mL and 80% motility were used for IVF. For IVF, the oocytes were transferred into 500 µL IVF medium (Tyrode’s lactate medium supplemented with 0.5 mM sodium pyruvate, 0.5 mM GlutaMAX (GlutaMAX™, Gibco® (Merck), Darmstadt, Germany), 2 mM CaCl_2_.2H2O, 1% v/v minimum nonessential amino acid solution (NEAA 100x, PAN Biotech, Aidenbach, Germany), 0.3% wt/vol BSA, 10 IU/mL penicillin, and 10 µg/mL streptomycin (Penicillin–Streptomycin, PAN Biotech, Aidenbach, Germany) under oil (37.5 °C, 5% O_2_/5.5% CO_2_). Sperm concentration was adjusted to 300.000 sperm/mL.

### CRISPR injection and embryo culture

The following morning, 15–16 h after the sperm suspension was added into IVF drops, cumulus cells were removed by pipetting. Denuded oocytes were assessed for the presence of two polar bodies (PBs) and pronuclei (pn) and zygotes were injected with CRISPR/Cas9 to induce genetic modification (results will be published separately). All fertilized oocytes analysed in the present study received injection of CRISPR/Cas9, therefore no additional variation in the study groups was introduced by this manipulation. In brief, at the pronuclear stage, intracytoplasmic injection of CRISPR/Cas9 mixture (4 × 15 ng/μL sgRNAs, 100 ng/μL of Cas9 protein (NEB, Ipswich, USA), 25 ng/μL of hCas9 mRNA, 5 mM TrisHCL pH 7.4 buffer and 100 mM NaCl) was performed. The mixture was injected using 1.6 µm pronucleus injection pipettes (Type PI-1.6, BioMedical Instruments, Zöllnitz, Germany) attached to the FemtoJet 4i microinjector (Eppendorf, Hamburg, Germany). Immediately after CRISPR/Cas9 injection, zygotes were transferred into culture plates with 50 µL ORIGIO® Sequential Cleav™ (Origio, Måløv, Denmark). Starting from the 4-cell stage, embryos were cultured in ORIGIO® Sequential Blast™ (Origio, Måløv, Denmark) at 37.5 °C, 5% O_2_/5.5% CO_2_.

From the morula stage (12-cell stage or initiation of compaction), ORIGIO® Sequential Blast™ was additionally supplemented with 2.5% FBS. Throughout the embryo culture period, every two days the embryos were transferred into new culture 4-well dishes with fresh medium chosen according to the developmental stage. Pictures of all cultured embryos were taken daily, with detailed recording of the developmental stage.

### Statistics

The analysis was performed using GraphPad Prism version 8.0 for Windows (GraphPad Software, Boston, Massachusetts USA, (www.graphpad.com). Differences between two groups were analysed using Student t-test for normally distributed samples; comparison of multiple groups was performed using one-way ANOVA with post-hoc Tukey multiple comparisons test. For evaluation of the relationships between variables, Pearson’s correlation coefficient was used. The results of correlation analysis are presented in the form of correlation coefficients and *p*-values. Differences were considered significant at *p* < 0.05, otherwise the differences are labelled as ‘ns’ (not significant) in the text. Unless indicated otherwise, the data are presented as mean ± SD.

## Results

### Oocyte recovery

**Fig. 2:**
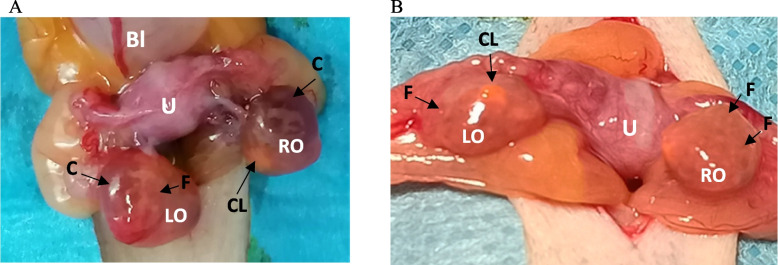
Morphological appearance of marmoset monkey ovaries after stimulation with FSH + hCG (**A**) and after FSH-priming (**B**). The ovaries stimulated with FSH + hCG visually appear larger (no measurements were performed due to sterility considerations), with presence of cysts and a high degree of hyperaemia (**A**). In contrast, the ovaries after FSH-priming have lighter colour and have evenly distributed large antral follicles (**B**). Bl – bladder; U – uterus, C – cyst, CL – *Corpus luteum*, RO – right ovary, LO – left ovary, F – follicle

After hormonal stimulation with FSH only (FSH-priming), ovaries appeared smaller, compared to the standard FSH + hCG protocol. This was due to the absence of large preovulatory follicles and no or lower number of cysts, which are a known consequence of hCG administration in women [44] (Fig. 2). No ovary measurements were performed during OPU or after ovariohysterectomy to avoid compromising sterility or delaying the processing. The risk of ovarian adhesions due to multiple OPUs was significantly reduced after FSH-priming compared to the standard FSH + hCG protocol. This might be attributed to lesser bleeding, which is one of the risk factors for adhesion formation [45]). Our main concern was whether it will be technically possible to recover at least the same number of oocytes following the FSH-priming protocol as following the standard stimulation protocol with hCG. Therefore, the number of the oocytes recovered from large ovulatory follicles was compared between the two study groups for each type of the oocyte recovery procedure (OPU1-3 and OvH).

**Fig. 3:**
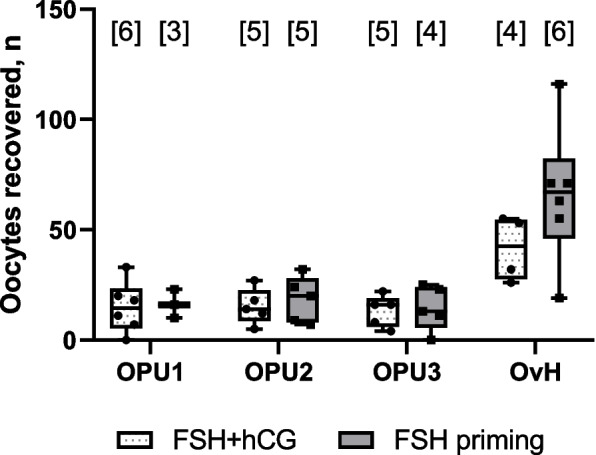
Total number of the non-degenerated oocytes recovered from large antral follicles in operations #1–4. For each animal, operations 1–3 were OPU (ovum pick-up); operation 4 was ovariohysterectomy (OvH). The number of stimulations of each type is shown in square brackets

For the purposes of statistical analysis, the data for OPUs 1–3 were pooled to compare the mean recovery rate after OPU between the two study groups. With the exception of one single case in the group of FSH + hCG, in all other stimulated cycles oocytes were successfully recovered. In total, following FSH + hCG stimulation, 14.4 ± 8.8 non-degenerated oocytes (incl. both immature and mature) were recovered per one OPU procedure vs. 16.4 ± 9.0 following FSH-priming (ns). Similarly, the recovery rate after ovary dissection upon OvH was at least as efficient with the stimulation approach without hCG, as with it (41.5 ± 14.7 in FSH + hCG group vs. 65.8 ± 31.3 FSH-priming group, ns) (Fig. 3).

### Oocyte maturation

#### In vivo matured

**Fig. 4:**
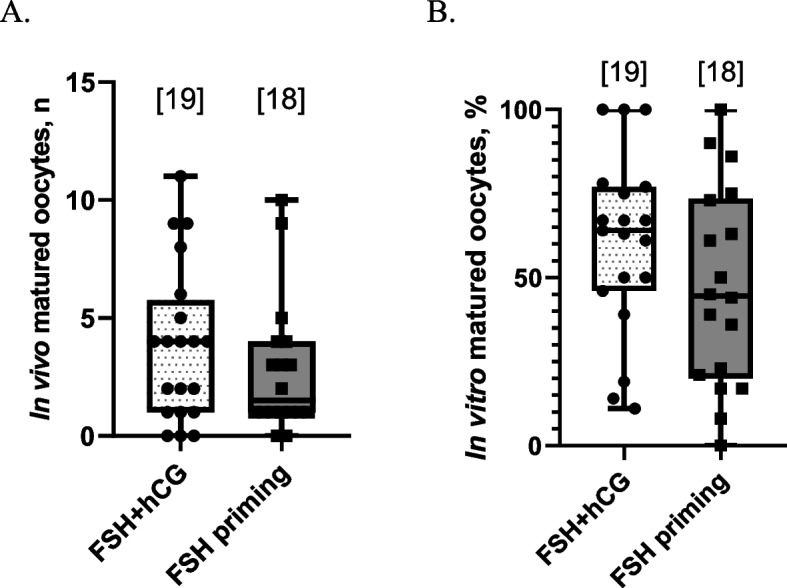
In vivo and in vitro maturation rates in the groups stimulated with FSH + hCG or FSH-priming. **A** Number of in vivo matured oocytes recovered from large antral follicles by aspiration or during follicle dissection. The number of stimulations in each group is shown in square brackets. **B** in vitro maturation rate from the total number of immature oocytes recovered. The number of stimulations in each group is shown in square brackets. The single case where no oocytes were recovered (FSH + hCG group) was not included in this analysis

Following FSH + hCG stimulation, the mean number of in vivo matured MII oocytes collected in each experiment was 3.9 ± 3.3. Surprisingly, this number was not significantly lower after FSH-priming (2.8 ± 2.8, ns) (Fig. 4A). In addition, there was no difference in the number of in vivo matured oocytes between OPU1, OPU2, OPU3 and OvH and MII number varied from 0 to 11 in the FSH + hCG group and from 0 to 10 in the FSH-priming group (ns). Therefore, for graphical presentation, data from all operation types were pooled for each study group (Fig. 4A).

#### In vitro matured

In total, 320 immature oocytes were recovered from 20 FSH + hCG stimulations and 558 immature oocytes were recovered from 18 FSH-priming experiments. The two study groups did not differ in the rates of in vitro oocyte maturation: 60.4 ± 26.7% oocytes reached the MII stage in the FSH + hCG group during IVM culture and 47.1 ± 29.8% in the FSH-priming group (ns) (Fig. 4B).

**Fig. 5:**
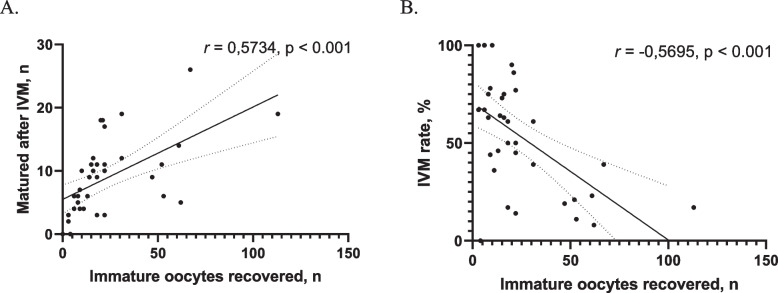
Correlation between the total number of recovered immature oocytes and the IVM outcome. This was represented either by the number of oocytes that successfully matured in vitro (**A**), or as the percentage of those matured in vitro from the total number of immature oocytes (**B**). The graphs show individual value data points, best-fit regression lines (solid lines), upper and lower 95% confidence intervals (dotted lines), and the respective Pearson correlation coefficients

While, as expected, there was a positive correlation between the number of recovered oocytes and the actual number of those which later matured to MII stage in vitro (*r* = 0.5734, *p* < 0.001, Fig. 5A), the number of recovered oocytes negatively correlated with the proportion of oocytes matured to MII stage in vitro (*r* = -0.5695, *p* < 0.001, Fig. 5B). This suggests that with the increasing numbers, a greater part of those recovered was developmentally incompetent.

**Fig. 6:**
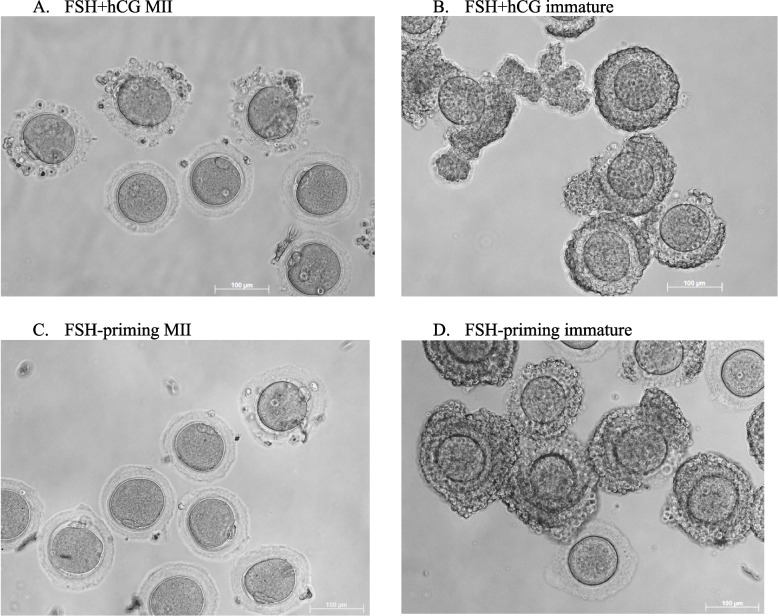
Morphology of oocytes recovered as in vivo matured MII oocytes or immature oocytes. The recovered MII oocytes (**A**, **C**) sometimes were either surrounded by expanded *corona radiata* cells or naked. The recovered immature oocytes (**B**, **D**) were either surrounded by several compact granulosa cell layers or were naked. No significant morphological differences were seen between either in vivo matured oocytes recovered from FSH + hCG stimulated (**A**) or FSH-primed ovaries (**C**); or immature oocytes (**B** vs. **D**, respectively)

Morphological appearance of mature and immature recovered oocytes was similar between both study groups (Fig. 6 A-D).

## IVF

**Fig. 7:**
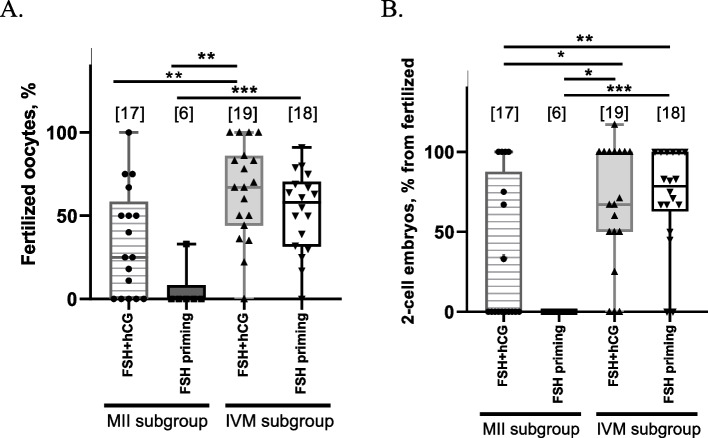
In vitro fertilization and first cleavage rates. **A** In ‘FSH + hCG, MII group’ and ‘FSH-priming, MII group’ the fertilization rates were calculated from the number of in vivo matured oocytes. In ‘FSH + hCG, IVM group’ and ‘FSH-priming, IVM group’ the fertilization rates were calculated from the number of oocytes matured in vitro. **B** First cleavage rate calculated as the percent of 2-cell embryos from fertilized oocytes. Both MII groups refer to in vivo matured oocytes, while IVM group includes oocytes matured in vitro. The number of stimulations in each group is shown in square brackets. The number of stimulations in each group is shown in square brackets. The experiments during which no mature oocytes were obtained were not included in the respective groups. * *p* < 0.05, ** *p* < 0.01, *** *p* < 0.001

While no significant effect of the stimulation approach on fertilization rates was observed in this study, surprisingly, fertilization was significantly lower in both MII subgroups compared to the oocytes matured in vitro (*p* < 0.05). Following stimulation with FSH + hCG, fertilization rates were 34.5 ± 32.0% in the in vivo matured subgroup and 64.8 ± 28.8 in the IVM subgroup; following FSH-priming fertilization was seen in 5.5 ± 13.5% of in vivo matured oocytes and 52.3 ± 24.4% of IVM oocytes (see Fig. 7A for statistical differences).

### Embryo development

Same as with fertilization rates, the reduced quality of the in vivo matured oocytes was suggested by a significantly lower first cleavage rate in both MII subgroups compared to IVM subgroups (Fig. 7B). From the total number of fertilized oocytes, only 33.8 ± 44.5% in vivo matured oocytes underwent first cleavage in FSH + hCG group and no cleavage (0 ± 0.00%) was seen in the in vivo matured subgroup in the FSH-priming group. These rates were significantly different (*p* < 0.05) from those observed in the IVM subgroups, both of which had a similarly high percentage of 2-cell embryos (66.2 ± 38.2 and 71.7 ± 31.4, respectively) (Fig. 7B).

To better visualize the overall efficiency of each protocol, embryo formation rates were calculated as a total proportion of MII oocytes which, after fertilization, progressed to 2- and 8-cell embryo stage in different groups (Table 2). While only a very small proportion of the in vivo matured oocytes in the FSH + hCG group could develop at least until the 8 cell stage (6%, 5/77) and no embryos were obtained from in vivo matured oocytes in the FSH-priming group; the rates in the IVM subgroup were 21% (30/142) and 28% (54/195) in the FSH + hCG and FSH-priming groups, respectively (Table 2). Trying to choose between the two protocols for future projects, we were interested in the actual numbers of progressed embryos that could be obtained with each approach. As much lower numbers of oocytes can be recovered with OPU compared with OvH, these two types of operations were analysed separately. In this case, due to low numbers, ANOVA only showed differences in embryo production between MII and IVM subgroups only (see Supplementary Table 1). Therefore, we calculated the total outcome as the sum of the total embryos obtained in each group divided by the number of the respective experiments. In total, 1.7 eight-cell embryos/experiment (OPU) and 2.1 eight-cell embryos/experiment (OvH) were obtained after FSH/hCG stimulation vs. 1.8 eight-cell embryos/experiment (OPU) and 5.0 eight-cell embryos/experiment (OvH) following FSH-priming. These numbers include embryos obtained from both in vivo and in vitro matured oocytes (Table 2).

**Table 2:** Total embryo outcome per experiment for different study groups

	**In vivo matured oocytes**				**in vitro matured oocytes**			
	**FSH + hCG**		**FSH-priming**		**FSH + hCG**		**FSH-priming**	
**Total matured oocytes, n**	77		20		142		195	
**Rate and total numbers of 2-cell embryos from matured oocytes**	18% (14/77)		0% (0/20)		49% (70/142)		44% (86/195)	
**Rate and total numbers of 8-cell embryos from matured oocyte**	6% (5/77)		0% (0/20)		21% (30/142)		28% (54/195)	
**Total experiments, n**	20		6^a^		20		18	
**Operation type**	OPU	OvH	OPU	OvH	OPU	OvH	OPU	OvH
**Number of experiments by operation type (OPU/OvH)**	16	4	4^a^	2^a^	16	4	12	6
**Number of 8-cell embryos per experiment**	0.3	0.3	0	0	1.4	1.8	2.0	5.0

^a^In vivo matured oocytes were used for IVF and further culture only in 6 out of 18 FSH-priming experiments, as IVF attempts were terminated for the MII subgroup once it had become obvious that these oocytes are not developmentally competent

**Fig. 8:**
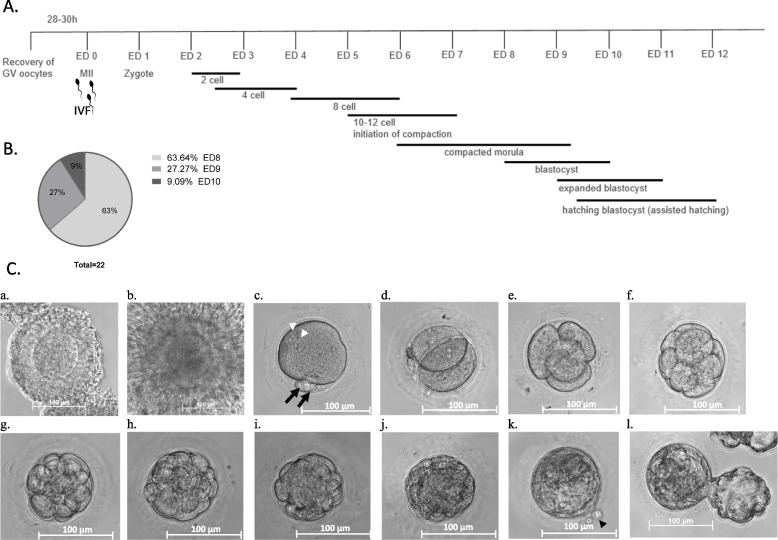
*Callithrix jacchus* embryo development in vitro and morphological appearance of the respective developmental stages. **A** Timeline of embryo development from in vivo matured oocytes (recovered at MII stage, starts from embryo day 0 (ED0) or in vitro matured oocytes (recovered at the germinal vesicle (GV) stage, starts from 28–30 h IVM). **B** Proportion of embryos with blastocoel first visible on ED8, ED9 and ED10 analysed in 22 blastocysts obtained during extended culture of IVM oocytes in FSH-priming group (*n* = 9). **C** Morphological appearance of the respective developmental stages: immature cumulus-oocyte complex (**a**); COC after IVM, with expanded cumulus surrounding the oocyte (**b**); fertilised oocyte with visible 2 PB’s (black arrows) and 2 pn (white arrowheads) (**c**); 2-cell embryo (**d**); 4-cell embryo (**e**); 8-cell embryos (**f**); 12-cell embryo (**g**), initiation of compaction (**h**); compacted morula (**i**); morula-blastocyst transition (**j**); blastocyst with a visible *zona pellucida* cut (black arrowhead) after artificial hatching performed at morula stage (**k**); hatching blastocyst (**l**)

In the FSH + hCG group, the embryos produced from MII oocytes were cultured until embryo day (ED) 6 and the embryos produced from IVM oocytes – until ED5 (the culture ended on a specific day of the week, i.e. when the embryo transfer to surrogate mothers was performed; embryo transfer results will be published separately). With the beginning of FSH-priming, we also extended the culture duration as a part of the protocol optimization, first until ED8 (embryos were produced only from IVM oocytes), later we removed the time limit to let the embryos that required longer than 8 days to form the blastocoel, to develop to this stage. Therefore, the data for blastocyst rates is only available for those nine FSH-priming experiments, where all potentially competent embryos were allowed to complete blastocyst formation. In general, blastocyst formation rate varied from 3 to 17% when calculated from the total number of recovered immature oocytes and from 6 to 40% of the in vitro matured (Table 3). The timescale of marmoset embryo development in vitro and the morphological appearance of the respective stages is shown in Fig. 8A, C.

**Table 3:** Individual blastocyst rates calculated from the total number of immature oocytes obtained after FSH-priming

Experiment #	OP type	Total immature oocytes recovered, n	MII after IVM	Blastocysts, n	Blastocyst rate from recovered immature oocytes, %	Blastocyst rate from IVM MII, %
1	OvH	62	5	2	3%	40%
2	OPU	15	11	2	13%	18%
3	OPU	22	10	1	5%	10%
4	OPU	6	6	1	17%	17%
5	OPU	20	18	1	5%	6%
6	OvH	113	19	4	4%	21%
7	OvH	67	26	7	10%	27%
8	OvH	52	11	2	4%	18%
9	OPU	11	4	1	9%	25%

The data are shown for the nine experiments, where immature oocytes recovered following FSH-priming, underwent maturation, fertilization and long culture until blastocyst stage

*OPU* Ovum pick-up, *OvH* Ovariohysterectomy

Most embryos capable of the development to the blastocyst stage formed a blastocoele at ED 8 (63%) (Fig. 8B). The latest blastocoele formation was observed on ED10, but all these were of suboptimal quality (poor morphology and or low number of trophectoderm and/or inner cell mass (ICM) cells).

## Discussion

Although several protocols of ovarian stimulation have been published for the common marmoset [26, 28, 29, 32, 34, 46, 47], the reported outcomes can often not be achieved in other research centres adopting the same stimulation scheme. Among other factors, this might be attributable to the differences in phenotype and/or genotype of the animals from different breeding colonies [48]. Since the implementation of Brazil’s restrictions on marmoset export in 1974, only a very small number of new *C. jacchus* individuals have entered captive colonies worldwide [49, 50]. As a result, multiple phenotypical and physiological changes have accumulated in those closed populations. Of these, the easiest to observe is body weight, which varies significantly in different colonies [51–53] and is strongly associated with the quality of the ovaries and the follicular reserve in the marmoset [54].

All these factors may potentially affect the animal's response to hormonal stimulations and may explain why the protocol developed by one laboratory may not always suit animals originating from a different colony. Moreover, similar to the situation seen in assisted human reproduction, no standard protocol can be reliably applied even within the same colony, since inter-individual differences need to be considered. Therefore, our primary goal was to individualize the stimulation conditions to maximize the oocyte harvest and embryo production. To this end, the duration of FSH stimulation and the dose of FSH (25–35 IU) was adjusted for each animal, as described above (see Materials and Methods).

It is also noteworthy that human gonadotropin hormones are used in primate reproductive research due to the lack of the respective species-specific preparations, and these may be a suboptimal substitute. Most published studies [26, 28, 55] used hFSH at the dose of 25–50 IU/animal. Originally, Marshall et al. [28] have shown that only 50 IU of hFSH, corresponding to approx. 100–150 IU/kg, increased the number of dominant follicles in marmosets. This dose is significantly higher than that usually used for ovarian stimulation in women (2–5 IU/kg) [56–58]. In marmosets, such low doses of hFSH (6 IU/kg) produced only a slight shift in the follicle population towards larger sizes [32]. Similarly, in rhesus higher doses of hFSH are usually reported for hormonal stimulation (10–20 IU/kg) [59, 60], although lower doses (3.5 IU/kg) may also produce sufficient ovarian response in this species [59–61]. Generally, lower efficiency of human FSH preparations in primates is likely attributed to species-unique specificity of gonadotrophins, which may be particularly true for marmosets, with their different type of the LH receptor [19] and not yet well studied reproductive endocrinology. As has already been suggested by other primate biologists [62], the use of primate-specific gonadotropin preparations would allow to perform more focused hormonal stimulation and possibly improve both the outcome and the quality of the oocytes and embryos.

In this study, we compared the efficacy of two hormonal stimulation approaches (hFSH stimulation with and without hCG) in the common marmoset monkey. The aim of the hCG injection at the final stage of the ovarian stimulation is to trigger the in vivo maturation of the oocytes from large antral follicles. However, here we observed the superior quality of the in vitro matured oocytes over those matured in vivo, indicating that in our settings the hCG injection step is unnecessary.

In humans, in vitro matured oocytes were shown to have higher rates of chromosomal and spindle defects and are generally less developmentally competent compared to those reaching the MII stage in vivo [63]. In contrast, in marmoset oocytes matured in vivo after hormonal stimulation with 50 IU hFSH for 9 days and 50 IU hCG on day 10 showed abnormal cytoskeletal formation [46], which supports our current findings. However, such discrepancy with the results published for humans suggests rather the inadequacy of the current protocols used for ovarian stimulation in the common marmoset than the intrinsically poorer quality of the oocytes matured in vivo after hCG triggering in this animal species. In support of this idea, in unstimulated cycles in marmosets after in vivo maturation triggering with hCG (75 IU) mature oocytes were derived from the naturally occurring dominant follicles, and all fertilized oocytes cleaved and 30% of the resulting embryos developed to blastocysts, showing excellent developmental competence [64]. In addition, in rhesus monkeys, a significant (30–40%) proportion of peri-ovulatory follicles displayed features of atresia after hCG injection [62], which compromised the quality of the oocytes derived from these follicles. This phenomenon may be related to a predetermined fate of dominant vs. subordinate follicles and warrants further investigation.

Another explanation of the better quality of the in vitro vs. in vivo matured oocytes in our study may be related to the age of the animals used (the mean age of the oocyte donors here was 4.6 years). The age at the time of the oocyte recovery is associated not only with the available follicular reserve, but also with the quality of the oocytes. Although efficient reproduction starts in marmoset females at around 2 years of age and markedly decreases only at 8–10 years of age [65, 66], differences in the ovarian morphology and in vitro embryo production outcomes can be seen in marmosets between 2 and 4 years of age [64]. A very recent human study showed that with the advancing age, although the number of the recovered oocytes naturally decreases, the oocytes collected at the GV stage become more developmentally competent, while the quality of the in vivo matured oocytes deteriorates [36, 67]. The differences shown for women between 32 and 40 years of age and those over 45 years were dramatic: while for in vivo matured oocytes embryo production rate decreased from 64 to 27%, for IVM oocytes it increased from 2 to 50% [36, 67]. Aligning reproductive age between humans and marmosets poses challenges, yet this new data is worth of further research in primates.

Molecular analysis of freshly isolated in vivo and of in vitro matured oocytes obtained from both stimulation protocols, as well as a detailed biochemical analysis of blood serum during and following stimulation could reveal the differences in parameters and help understand the reasons for a diminished quality of in vivo matured marmoset oocytes. Unfortunately, such investigations were not possible in the present study, which has been performed within the framework of a larger project, due to restrictions imposed by the current animal experiment permission. We suggest further investigation in this direction.

In our study, no differences were observed between the two approaches in regard to the number of COCs recovered after OPUs or after OvHs. However, there might be a trend towards a larger number of COCs harvested after FSH-priming. Despite trying to select only those COCs which contained the oocytes of at least 80 µm in diameter (without the z*ona pellucida*, ZP) for IVM culture, we could not precisely measure the diameters of the oocytes while enclosed in cumulus cell layers. Therefore, the size-selection of immature COCs was only approximate. After hCG application, hyperaemia of the ovaries was routinely observed, which might be similar to the ovarian hyperstimulation syndrome (OHSS) in women, the risk of which increases significantly by hCG injection [36]. In contrast, in the FSH-priming group, the risk of bleeding and the subsequent formation of adhesions on the ovarian surface during follicle puncture was significantly lower. It is therefore possible that in the FSH-priming group, with more efficient follicle aspiration during OPU due to better visibility of the follicles or with a higher number of available large antral follicles punctured during ovary dissection, a higher number of smaller, not developmentally competent oocytes recovered from smaller sized follicles were selected for culture, compared to FSH + hCG stimulation. This assumption is further supported by the correlation analysis results, suggesting that the number of recovered oocytes was negatively associated with the number of developmentally competent oocytes, as their proportion from the total number of the recovered oocytes decreased with the increasing recovery rates.

Surprisingly, after elimination of maturation triggering with hCG, there were still MII oocytes collected from follicles following the FSH-priming only, and their number was not significantly lower than that recovered in the FSH + hCG group. A premature surge of a gonadotropic hormone exerting luteinizing functions (which is CG in case of the marmoset, as was already mentioned in the Introduction) could explain the presence of matured oocytes on the day of surgery. In marmosets, the ovulation can be timed by the measurement of serum progesterone (P_4_) concentration, with concentrations exceeding 10 ng/mL indicating that ovulation occurred [9, 41]. The levels of P_4_ < 10 ng/mL at the time of operation, however, cannot exclude that oocyte maturation has already taken place within the follicle. Further shortening of the FSH-stimulation duration may be beneficial, but the fact that MII oocytes were recovered in nearly 90% of FSH-stimulated cycles (16/18) strongly suggests that FSH overstimulation in terms of the timing was unlikely. Another issue that warrants further research is whether hrFSH injections may promote meiotic resumption of marmoset oocytes in vivo. In humans and rodents, FSH alone is shown to trigger oocyte maturation in vitro [68–70], but whether it could stimulate in vivo meiotic resumption in marmosets (e.g. mediated by EGF-like peptides) remains unknown. Further, whether the presence of the in vivo matured oocytes is associated with a poorer quality of the remaining immature ones is not clear, but, as mentioned above, immature oocytes in both stimulation groups still possessed a better quality in terms of embryo development rates compared to the ones recovered at the MII stage.

Total outcome of 8-cell embryos from both, in vivo and in vitro matured oocytes, was not different between the two protocols or even tended to be higher after OvH with FSH-priming compared to FSH + hCG (5.0 vs. 2.1). This convinced us to choose the FSH-priming protocol for further studies as having at least equal or even superior efficacy under our experimental settings of embryo production. Both protocols produced viable, developmentally competent embryos from in vitro (but not in vivo) matured oocytes. This was confirmed by pregnancies/offspring obtained after transfer of embryos from both stimulation protocols (pregnancies from FSH-priming stimulations are still ongoing, the data will be published separately).

All oocytes in this study were injected with CRISPR/Cas9 to generate genomic modification (the data will be reported separately). This should be taken into account when interpreting the outcome rates, as both the injection alone and the compounds injected are traumatic for the oocytes. With our FSH-priming protocol, 20% of the oocytes which reached MII following IVM progressed to blastocyst stage in culture (see Supplementary Table 2). This current rate for in vitro matured marmoset oocytes appears to even exceed the rate seen in in vivo matured (following hormonal stimulation) rhesus oocytes subject to similar manipulations: only 6 to 9% of rhesus MII oocytes injected with CRISPR/Cas9 mix at the zygote stage formed blastocysts [71]. In actual numbers, we have obtained between 1 and 7 blastocysts per experiment (mean 2.3/stimulation cycle). Comparing this outcome with the results of other laboratories working with marmosets proves challenging, since the reported data sometimes do not allow to exactly track the numbers to assess the efficiency of each operation/step of the protocol, especially in the presence of an additional stress factor, such as CRISPR/Cas9 injection. In older reports, similar marmoset blastocyst production rates of around 2.5 blastocysts/stimulation cycle were obtained upon application of various stimulation protocols with either hFSH alone or in combination with hCG, although in the absence of any genetic manipulations [25, 26, 28]. Recently, the number of zygotes but not blastocysts obtained following hormonal stimulation in marmosets were reported by Kishimoto et al*.* [27] being 4.98 per ovarian stimulation cycle, which is comparable with our rate of 5.6 zygotes/cycle after IVM (average for both IVM subgroups, data not shown).

In summary, in our settings, the FSH-priming protocol provides an outcome comparable to that reported by other labs and demonstrates at least equal or even better efficiency compared to FSH + hCG stimulation. In addition, FSH-priming reduces the burden on the animals by shortening the stimulation cycle (number of injections) and eliminates possible adverse effects of hCG. Furthermore, ovarian follicle puncture is generally easier and less harmful in FSH-primed animals due to a less pronounced ovarian hyperaemia and, consequently, reduced risk of intraoperative bleeding and postoperative adhesions. In view of the impaired quality of the in vivo matured oocytes compared to IVM ones, further studies aiming at an optimization of hormonal stimulation and culture conditions in marmoset are needed. Such protocols could help address the requirements for the in vivo development of dominant vs. subordinate follicles as well as the in vitro development of the oocytes recovered from them.

### Supplementary Information

Supplementary Material 1.

Supplementary Material 2.

## Data Availability

No datasets were generated or analysed during the current study.

## References

[1] Miller C, Mitchell J (2023). Marmoset Community White Paper 2023.

[2] Han HJ, Powers SJ, Gabrielson KL (2022). The Common Marmoset-Biomedical Research Animal Model Applications and Common Spontaneous Diseases. Toxicol Pathol.

[3] Power ML, Adams J, Solonika K, Colman RJ, Ross C, Tardif SD (2019). Diet, digestion and energy intake in captive common marmosets (Callithrix jacchus): research and management implications. Sci Rep.

[4] Tardif SD, Smucny DA, Abbott DH, Mansfield K, Schultz-Darken N, Yamamoto ME (2003). Reproduction in captive common marmosets (Callithrix jacchus). Comp Med.

[5] Drummer C, Vogt EJ, Heistermann M, Roshani B, Becker T, Mätz-Rensing K, et al. Generation and Breeding of EGFP-Transgenic Marmoset Monkeys: Cell Chimerism and Implications for Disease Modeling. Cells. 2021;10(3). 10.3390/cells10030505 PMC7996964 33673402

[6] Tomioka I, Ishibashi H, Minakawa EN, Motohashi HH, Takayama O, Saito Y, et al. Transgenic monkey model of the polyglutamine diseases recapitulating progressive neurological symptoms. eneuro. 2017;4(2). 10.1523/ENEURO.0250-16.2017 PMC5368386 28374014

[7] Dang D, editor Absence of seasonal variation in the length of the menstrual cycle and the fertility of the crab-eating macaque (Macaca fascicularis) raised under natural daylight ratio. Annales de Biologie Animale Biochimie Biophysique; 1977: EDP Sciences.

[8] Harding RD, Hulme MJ, Lunn SF, Henderson C, Aitken RJ (1982). Plasma progesterone levels throughout the ovarian cycle of the common marmoset (Callithrix jacchus). J Med Primatol.

[9] Harlow CR, Gems S, Hodges LK, Hearn JP (1983). The relationship between plasma progesterone and the timing of ovulation and early embryonic development in the marmoset monkey (Callithrix jacchus). J Zool (London).

[10] Bull JR, Rowland SP, Scherwitzl EB, Scherwitzl R, Danielsson KG, Harper J (2019). Real-world menstrual cycle characteristics of more than 600,000 menstrual cycles. npj Digital Med.

[11] Muter J, Lynch VJ, McCoy RC, Brosens JJ. Human embryo implantation. Development. 2023;150(10). 10.1242/dev.201507 PMC10281521 37254877

[12] Enders AC (2007). Implantation in the Macaque: Expansion of the Implantation Site During the First Week of Implantation. Placenta.

[13] Moore HD, Gems S, Hearn JP (1985). Early implantation stages in the marmoset monkey (Callithrix jacchus). Am J Anat.

[14] O'Rahilly R, Müller F (2010). Developmental stages in human embryos: revised and new measurements. Cells Tissues Organs.

[15] Heuser CH, Streeter GL (1941). Development of the macaque embryo.

[16] Phillips IR (1976). The embryology of the common marmoset (Callithrix jacchus). Adv Anat Embryol Cell Biol.

[17] Gromoll J, Wistuba J, Terwort N, Godmann M, Müller T, Simoni M (2003). A New Subclass of the Luteinizing Hormone/Chorionic Gonadotropin Receptor Lacking Exon 10 Messenger RNA in the New World Monkey (Platyrrhini) Lineage1. Biol Reprod.

[18] Fiddes JC, Goodman HM (1981). The gene encoding the common alpha subunit of the four human glycoprotein hormones. J Mol Appl Genet.

[19] Müller T, Simoni M, Pekel E, Luetjens CM, Chandolia R, Amato F (2004). Chorionic gonadotrophin beta subunit mRNA but not luteinising hormone beta subunit mRNA is expressed in the pituitary of the common marmoset (Callithrix jacchus). J Mol Endocrinol.

[20] Abbott DH, Hodges JK, George LM (1988). Social status controls LH secretion and ovulation in female marmoset monkeys (Callithrix jacchus). J Endocrinol.

[21] Chambers PL, Hearn JP (1979). Peripheral plasma levels of progesterone, oestradiol-17 beta, oestrone, testosterone, androstenedione and chorionic gonadotrophin during pregnancy in the marmoset monkey. Callithrix jacchus J Reprod Fertil.

[22] Harlow CR, Hearn JP, Hodges JK (1984). Ovulation in the marmoset monkey: endocrinology, prediction and detection. J Endocrinol.

[23] Ziegler TE, Matteri RL, Wegner FH (1993). Detection of urinary gonadotropins in callitrichid monkeys with a sensitive immunoassay based upan a unique monoclonal antibody. Am J Primatol.

[24] Gilchrist RB, Nayudu PL, Nowshari MA, Hodges JK (1995). Meiotic competence of marmoset monkey oocytes is related to follicle size and oocyte-somatic cell associations. Biol Reprod.

[25] Gilchrist RB, Wicherek M, Heistermann M, Nayudu PL, Hodges JK (2001). Changes in follicle-stimulating hormone and follicle populations during the ovarian cycle of the common marmoset. Biol Reprod.

[26] Grupen CG, Gilchrist RB, Nayudu PL, Barry MF, Schulz SJ, Ritter LJ, Armstrong DT (2007). Effects of ovarian stimulation, with and without human chorionic gonadotrophin, on oocyte meiotic and developmental competence in the marmoset monkey (Callithrix jacchus). Theriogenology.

[27] Kishimoto K, Shimada A, Shinohara H, Takahashi T, Yamada Y, Higuchi Y (2021). Establishment of novel common marmoset embryonic stem cell lines under various conditions. Stem Cell Res.

[28] Marshall VS, Browne MA, Knowles L, Golos TG, Thomson JA (2003). Ovarian stimulation of marmoset monkeys (Callithrix jacchus) using recombinant human follicle stimulating hormone. J Med Primatol.

[29] Takahashi T, Hanazawa K, Inoue T, Sato K, Sedohara A, Okahara J (2014). Birth of healthy offspring following ICSI in in vitro-matured common marmoset (Callithrix jacchus) oocytes. PLoS ONE.

[30] Kropp J, Di Marzo A, Golos T (2017). Assisted reproductive technologies in the common marmoset: an integral species for developing nonhuman primate models of human diseases. Biol Reprod.

[31] Park JE, Sasaki E (2020). Assisted Reproductive Techniques and Genetic Manipulation in the Common Marmoset. ILAR J.

[32] Gilchrist RB, Nayudu PL, Hodges JK (1997). Maturation, fertilization, and development of marmoset monkey oocytes in vitro. Biol Reprod.

[33] Kurotaki Y, Sasaki E (2017). Practical reproductive techniques for the common marmoset. J Mammalian Ova Res.

[34] Park JE, Zhang XF, Choi SH, Okahara J, Sasaki E, Silva AC (2016). Generation of transgenic marmosets expressing genetically encoded calcium indicators. Sci Rep.

[35] Sato K, Oiwa R, Kumita W, Henry R, Sakuma T, Ito R (2016). Generation of a nonhuman primate model of severe combined immunodeficiency using highly efficient genome editing. Cell Stem Cell.

[36] Nastri CO, Ferriani RA, Rocha IA, Martins WP (2010). Ovarian hyperstimulation syndrome: pathophysiology and prevention. J Assist Reprod Genet.

[37] Isachenko EF, Nayudu PL, Isachenko VV, Nawroth F, Michelmann HW (2002). Congenitally caused fused labia in the common marmoset (Callithrix jacchus). J Med Primatol.

[38] Tkachenko OY, Delimitreva S, Isachenko E, Valle RR, Michelmann HW, Berenson A, Nayudu PL (2010). Epidermal growth factor effects on marmoset monkey (Callithrix jacchus) oocyte in vitro maturation, IVF and embryo development are altered by gonadotrophin concentration during oocyte maturation. Hum Reprod.

[39] French JA, Mustoe AC. Sexual Behavior in Marmosets in the Context of Cooperative Breeding. In: Shackelford TK, editor. The Cambridge Handbook of Evolutionary Perspectives on Sexual Psychology: Volume 4: Controversies, Applications, and Nonhuman Primate Extensions. Cambridge Handbooks in Psychology. 4. Cambridge: Cambridge University Press; 2022. p. 464–93.

[40] Heistermann M, Tari S, Hodges JK (1993). Measurement of faecal steroids for monitoring ovarian function in New World primates. Callitrichidae J Reprod Fertil.

[41] Daskalaki M, Drummer C, Behr R, Heistermann M (2022). The use of alfaxalone for short-term anesthesia can confound serum progesterone measurements in the common marmoset: a case report. Primate Biol.

[42] Tkachenko OY, Delimitreva SM, Wedi E, Scheerer-Bernhard JU, Valle RR, Nayudu PL (2017). Effects of oxygen concentration in IVM/IVF on marmoset monkey oocyte maturation and embryo development. Anim Reprod.

[43] Valle RR, Valle CM, Nichi M, Muniz JA, Nayudu PL, Guimaraes MA (2008). Validation of non-fluorescent methods to reliably detect acrosomal and plasma membrane integrity of common marmoset (Callithrix jacchus) sperm. Theriogenology.

[44] Tummon IS, Henig I, Radwanska E, Binor Z, Rawlins R, Dmowski WP (1988). Persistent ovarian cysts following administration of human menopausal and chorionic gonadotropins: an attenuated form of ovarian hyperstimulation syndrome. Fertil Steril.

[45] Wallwiener M, Brölmann H, Koninckx PR, Lundorff P, Lower AM, Wattiez A (2012). Adhesions after abdominal, pelvic and intra-uterine surgery and their prevention. Gynecol Surg.

[46] Kanda A, Nobukiyo A, Yoshioka M, Hatakeyama T, Sotomaru Y (2018). Quality of common marmoset (Callithrix jacchus) oocytes collected after ovarian stimulation. Theriogenology.

[47] Sotomaru Y, Hirakawa R, Shimada A, Shiozawa S, Sugawara A, Oiwa R (2009). Preimplantation development of somatic cell cloned embryos in the common marmoset (Callithrix jacchus). Cloning Stem Cells.

[48] Anestidou L, Johnson AF. Care, Use, and Welfare of Marmosets as Animal Models for Gene Editing-Based Biomedical Research: Proceedings of a Workshop: The National Academies Press; 2019. 31381285

[49] CITES Trade Database. Version 2023.1. Compiled by UNEP-WCMC, Cambridge, UK for the CITES Secretariat, Geneva, Switzerland. Available at: trade.cites.org 2023 [

[50] de Souza FM, Ludwig G, Valença-Montenegro MM (2016). Legal international trade in live neotropical primates originating from South America. Primate Conservation.

[51] Cawthon Lang KA. Primate Factsheets: Common marmoset (Callithrix jacchus) Taxonomy, Morphology & Ecology 2005 [Available from: http://pin.primate.wisc.edu/factsheets/entry/common_marmoset.

[52] Tardif SD, Power ML, Ross CN, Rutherford JN, Layne-Colon DG, Paulik MA (2009). Characterization of obese phenotypes in a small nonhuman primate, the common marmoset (Callithrix jacchus). Obesity (Silver Spring).

[53] Wedi E (2010). Untersuchung des CFL-Phänotyps ("congenital fused labia") in dem Neuweltaffen Common Marmoset (Callithrix jacchus) unter demographischen, physiologischen und zytogenetischen Gesichtspunkten.

[54] Scheerer-Bernhard JU, Tkachenko OY, Heistermann M, Gründker C, Nayudu PL (2015). Body weight-associated differences in ovarian morphology in captive common marmoset (Callithrix jacchus). Anim Reprod Sci.

[55] Sasaki E, Suemizu H, Shimada A, Hanazawa K, Oiwa R, Kamioka M (2009). Generation of transgenic non-human primates with germline transmission. Nature.

[56] Dorn C (2005). FSH: what is the highest dose for ovarian stimulation that makes sense on an evidence-based level?. Reprod Biomed Online.

[57] Fatemi H, Bilger W, Denis D, Griesinger G, La Marca A, Longobardi S (2021). Dose adjustment of follicle-stimulating hormone (FSH) during ovarian stimulation as part of medically-assisted reproduction in clinical studies: a systematic review covering 10 years (2007–2017). Reprod Biol Endocrinol.

[58] Lekamge DN, Lane M, Gilchrist RB, Tremellen KP (2008). Increased gonadotrophin stimulation does not improve IVF outcomes in patients with predicted poor ovarian reserve. J Assist Reprod Genet.

[59] Ramsey C, Hanna C (2019). In Vitro Culture of Rhesus Macaque (Macaca mulatta) Embryos. Methods Mol Biol.

[60] Zheng P, Si W, Wang H, Zou R, Bavister BD, Ji W (2001). Effect of age and breeding season on the developmental capacity of oocytes from unstimulated and follicle-stimulating hormone-stimulated rhesus monkeys. Biol Reprod.

[61] Yang S, He X, Hildebrandt TB, Zhou Q, Ji W (2007). Superovulatory response to a low dose single-daily treatment of rhFSH dissolved in polyvinylpyrrolidone in rhesus monkeys. Am J Primatol.

[62] Stouffer RL, Zelinski-Wooten MB (2004). Overriding follicle selection in controlled ovarian stimulation protocols: Quality vs quantity. Reprod Biol Endocrinol.

[63] Li Y, Feng HL, Cao YJ, Zheng GJ, Yang Y, Mullen S (2006). Confocal microscopic analysis of the spindle and chromosome configurations of human oocytes matured in vitro. Fertil Steril.

[64] Tkachenko OY (2012). In vitro oocyte maturation and embryo production in the common marmoset (Callithrix jacchus) [Dissertation Thesis].

[65] Smucny DA, Abbott DH, Mansfield KG, Schultz-Darken NJ, Yamamoto ME, Alencar AI, Tardif SD (2004). Reproductive output, maternal age, and survivorship in captive common marmoset females (Callithrix jacchus). Am J Primatol.

[66] Tardif SD, Araujo A, Arruda MF, French JA, Sousa MB, Yamamoto ME (2008). Reproduction and aging in marmosets and tamarins. Interdiscip Top Gerontol.

[67] Nicholas C, Darmon S, Patrizio P, Albertini DF, Barad DH, Gleicher N (2023). Changing clinical significance of oocyte maturity grades with advancing female age advances precision medicine in IVF. iScience.

[68] Cadenas J, Nikiforov D, Pors SE, Zuniga LA, Wakimoto Y, Ghezelayagh Z (2021). A threshold concentration of FSH is needed during IVM of ex vivo collected human oocytes. J Assist Reprod Genet.

[69] Gilchrist RB, Smitz J (2023). Oocyte in vitro maturation: physiological basis and application to clinical practice. Fertil Steril.

[70] Lin YH, Hwang JL, Seow KM, Huang LW, Hsieh BC, Chen HJ (2011). Effect of incubation with different concentrations and durations of FSH for in-vitro maturation of murine oocytes. Reprod Biomed Online.

[71] Ryu J, Statz JP, Chan W, Burch FC, Brigande JV, Kempton B (2022). CRISPR/Cas9 editing of the MYO7A gene in rhesus macaque embryos to generate a primate model of Usher syndrome type 1B. Sci Rep.

