# Supplementary material for: In vitro matured oocytes have a higher developmental potential than in vivo matured oocytes after hormonal ovarian stimulation in Callithrix jacchus
Source: J Ovarian Res. 2024 Jun 1;17:120. doi: 10.1186/s13048-024-01441-0 (PMC11144324; doi:10.1186/s13048-024-01441-0)
Supplement: Supplementary file 2 — Supplementary Material 2. [file 13048_2024_1441_MOESM2_ESM.docx]

**Experiments with an extended culture period (at least until blastocyst formation)**

|  | Experiment | Date | Animal ID | OP type | Total immature oocytes recovered | Blastocysts, n | Blastocyst rate from recovered immature, % | MII after IVM | Blastocysts from MII |
| --- | --- | --- | --- | --- | --- | --- | --- | --- | --- |
|  | 1 | 26.04.2023 | 17366 | OvH | 62 | 2 | 3% | 5 | 40% |
|  | 2 | 28.06.2023 | 17683 | OPU | 15 | 2 | 13% | 11 | 18% |
|  | 3 | 12.07.2023 | 17667 | OPU | 22 | 1 | 5% | 10 | 10% |
|  | 4 | 12.07.2023 | 17646 | OPU | 6 | 1 | 17% | 6 | 17% |
|  | 5 | 16.08.2023 | 17562 | OPU | 20 | 1 | 5% | 18 | 6% |
|  | 6 | 16.08.2023 | 17750 | OvH | 113 | 4 | 4% | 19 | 21% |
|  | 7 | 23.08.2023 | 17665 | OvH | 67 | 7 | 10% | 26 | 27% |
|  | 8 | 27.09.2023 | 17667 | OvH | 52 | 2 | 4% | 11 | 18% |
|  | 9 | 04.10.2023 | 17683 | OPU | 11 | 1 | 9% | 4 | 25% |
|  | TOTAL |  |  |  | 368 | 21 | 8% | 110 | 20% |
